# Recovery Potential After Acute Stroke

**DOI:** 10.3389/fneur.2015.00238

**Published:** 2015-11-11

**Authors:** Rüdiger J. Seitz, Geoffrey A. Donnan

**Affiliations:** ^1^Department of Neurology, Centre of Neurology and Neuropsychiatry, LVR-Klinikum Düsseldorf, Heinrich-Heine-University Düsseldorf, Düsseldorf, Germany; ^2^Biomedical Research Centre, Heinrich-Heine-University Düsseldorf, Düsseldorf, Germany; ^3^Florey Institute of Neuroscience and Mental Health, University of Melbourne, Parkville, VIC, Australia

**Keywords:** cerebral ischemia, infarct location, thrombolysis, recovery, perilesional plasticity, network reorganization, stroke associated disturbances, neurorehabilitative training

## Abstract

In acute stroke, the major factor for recovery is the early use of thrombolysis aimed at arterial recanalization and reperfusion of ischemic brain tissue. Subsequently, neurorehabilitative training critically improves clinical recovery due to augmention of postlesional plasticity. Neuroimaging and electrophysiology studies have revealed that the location and volume of the stroke lesion, the affection of nerve fiber tracts, as well as functional and structural changes in the perilesional tissue and in large-scale bihemispheric networks are relevant biomarkers of post-stroke recovery. However, associated disorders, such as mood disorders, epilepsy, and neurodegenerative diseases, may induce secondary cerebral changes or aggravate the functional deficits and, thereby, compromise the potential for recovery.

## Introduction

Stroke is one of the leading causes of persistent disability in Western countries (1). It induces acute deficits of motion, sensation, cognition, and emotion. In the majority of patients, stroke results from an interruption of cerebral blood supply and subsequent ischemic brain damage, while >25% of patients suffer from intracranial hemorrhage (2, 3). Recovery from stroke is a multifaceted process depending on different mechanisms that become operational at different phases after the acute insult ranging from hours to many months (4). Importantly, intravenous and intra-arterial thrombolyses have opened new avenues to substantially reverse the amount of brain damage and the neurological deficit after stroke (5–8). Furthermore, neuroscience-based strategies in neurorehabilitation have improved the fate of stroke patients. Specifically, training approaches including very early mobilization, antigravity support for walking, basic arm training, and arm ability training can be tailored to the neurological deficits to optimally engage the residual capacities of the patients (9–11). From a technical point of view, neuroimaging and neurophysiological methods have offered means to investigate the recovery potential of stroke patients already in the acute stage of stroke (12–14). In particular, these non-invasive neuroscientific measures substantiate clinical observations and have opened new insights into the neuroscientific basis of recovery mechanisms from stroke. More recently, the recovery potential after stroke has been studied by using multivariate analyses in which epidemiological factors have also been taken into account (15). We address here the mechanisms of post-stroke recovery including postlesional plasticity and disease-related limitations of the recovery potential in acute ischemic stroke.

## Mechanisms of Post-Stroke Recovery

### Dynamics of Cerebral Ischemia

**Figure 1:**
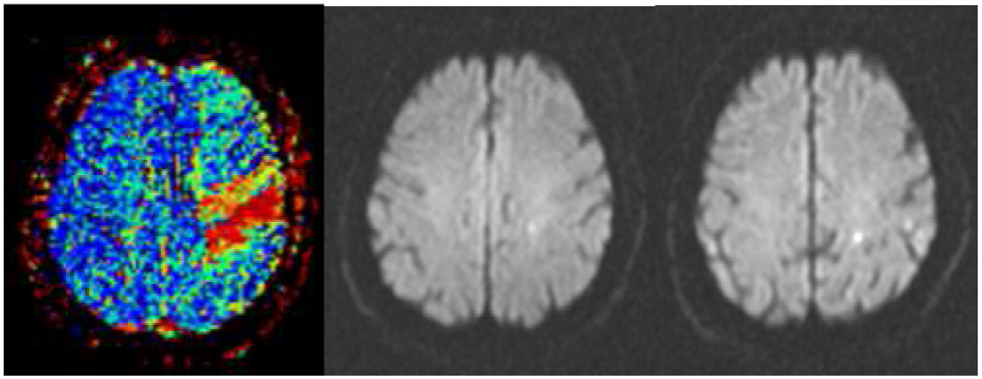
**Successful thrombolysis**. (Left) Severe perfusion deficit in the precentral gyrus (red) as assessed in a time-to-peak map before thrombolysis. (Middle) Point-like abnormality in diffusion-weighted imaging at the same time signifying the perfusion–diffusion mismatch. (Right) Two small lesions in diffusion-weighted imaging 24 h after intravenous thrombolysis accompanied by complete recovery from hemiparesis.

A sudden interruption of arterial blood supply leads to disturbances of neural function and the clinical appearance of neurological or neuropsychological deficits. In the most severe cases, ischemia is so severe that structural brain damage and the formation of ischemic brain infarction occur (Figure 1). The cessation of cerebral blood circulation induces an immediate suppression of cerebral electrical activity with peri-infarct depolarization leading to repeated episodes of metabolic stress (16, 17). There is good evidence from animal experiments that ischemic damage of neurons and brain tissue occurs in proportion to the reduction of regional cerebral blood flow (rCBF) (16). Thus, the acute occlusion of a cerebral artery, the thereby caused local depression of rCBF, and its subsequent electrical, metabolic, and ionic changes are critical factors determining the extent of a cerebral ischemic infarct (18). Imaging and neurophysiological studies in humans have shown that, similar to animal experiments, spreading depression occurs in severe ischemic stroke leading to progressive infarct expansion (19, 20).

After occlusion of a cerebral artery, an area of impaired perfusion surrounds an area with a complete cessation of perfusion whose extent is determined by the compensatory recruitment of arterial collaterals. In the area of misery perfusion, the so-called penumbra, the extraction of oxygen from blood into brain tissue is enhanced as was shown in stroke patients by multiparametric imaging with positron emission tomography (21, 22). The advent of magnetic resonance imaging (MRI) has allowed a spatial dimension to be introduced. It has been shown that the area of impaired perfusion typically exceeds the area of reduced extracellular water diffusion, thus signifying virtually reversible brain tissue damage due to ischemia (23–25). In fact, there is a good correspondence between the area with enhanced oxygen extraction and the perfusion–diffusion mismatch area in acute stroke (26, 27).

The area of reduced brain perfusion undergoes a dynamic lesion transformation within the first 24 h after onset of ischemia (28–30). In a persisting arterial occlusion, the infarct lesion expands up to 24 h (31, 32). Beyond the acute time window of about 24 h, secondary changes including an early phase with vasogenic edema and a later phase with inflammatory infiltration evolve (33–35). Lymphocytes and macrophages have been shown to accumulate in the perivascular vicinity ~6 days after a cerebral infarction and are heterogeneously distributed within the infarct area (36). Due to their immunological competence, these cells are suited to augment the infarct lesion raising the interesting notion that immunosuppression may have a beneficial affect in acute stroke (37).

### Reversal of Cerebral Ischemia

In acute ischemic stroke, intravenous thrombolysis is targeted toward the rescue of brain tissue by early recanalization of the occluded cerebral artery. It has been shown to be effective up to 4.5 h with maximal efficacy within the first 90 min after symptom onset (5, 6, 38). The beneficial role of early recanalization was demonstrated by functional brain imaging (39–42) and monitoring with transcranial Doppler sonography (43, 44). More recently, neuroradiological interventions with intra-arterial thrombolysis and/or thrombectomy have been shown to be at least as effective as intravenous thrombolysis even in distal carotid or proximal middle cerebral artery (MCA) occlusion (8). By multiparametric MRI, it became evident that brain tissue at the risk of ischemic damage can be salvaged by tissue reperfusion (Figure 1). Important factors determining the extent of a brain infarct are the severity and duration of ischemia, the dimension and composition of the causal arterial emboli, the anatomy and the vascular changes of the cerebral arteries, and the presence of diabetic hyperglycemia (29, 41, 45–47). In failed reperfusion, severe edema formation will develop that can hardly be limited pharmacologically. Thus, to rescue patients from malignant brain swelling after stroke craniectomy has been advocated as a symptomatic therapy which is a life-saving action but does not reduce the neurological deficit in patients older than 60 years (48).

Brain infarcts may result from cardiac or artery to artery embolism, from thrombotic occlusion of the small penetrating arteries complicating vessel hyalinosis or microatheroma (49, 50). While infarcts in the territory of the posterior cerebral artery (PCA) are typically embolic in origin affecting the entire supply area of the PCA (51), infarcts in the anterior cerebral artery (ACA) territory are usually of atherosclerotic origin and more variable in lesion pattern and neurological deficit (52). The situation is most complex in the MCA territory because of the arborization of the MCA, the large territory supplied by the artery, and the widespread anastomoses of the leptomeningeal arterial branches fed from the ACA or PCA. The poorer these collaterals are due to arterosclerotic changes in the intracranial arteries, the more severe is the initial ischemic event and the resulting stroke lesion (41, 53, 54).

The location and the volume of the cerebral infarct determine the neurological deficit in an individual patient as shown for sensorimotor as well as cognitive and emotional functions (55–61). Large brain infarcts involving subcortical white matter may affect multiple brain systems which may result in complex neurological syndromes, such as apraxia, neglect, and Gerstman’s syndrome (62–64). In such patients, measures of fiber tract damage or cortical activations have been found to predict the degree of recovery (55, 65–68). Similar observations have also been made for language, somatosensory and visual functions (69–72).

### Residual Brain Infarct Lesions After Thrombolysis

The successful recanalizing therapy is of fundamental importance for the topography and volume of the resulting ischemic infarct lesion (73, 74). This was taken into consideration in developing a refined classification of ischemic brain infarcts (75). It should be stated, however, that the functional prognosis of ischemic stroke is worse than that in cerebral hemorrhage in stroke survivors (76). This most likely reflects the structural damage of brain tissue in ischemic stroke, while in cerebral hemorrhage recovery can occur largely upon absorption of the hematoma. Accordingly, territorial Type I infarcts depend on the size of the emboli and the location of the arterial occlusion (Table 1). Distal arterial branch occlusion gives rise to small infarcts entirely limited to the cerebral cortex, while proximal arterial branch occlusions result in larger infarcts involving the cerebral cortex and the underlying white matter (77, 78). In MCA stroke, these territorial infarcts do not destroy the entire motor and somatosensory representation areas, nor the complete descending motor cortical output or afferent sensory input tracts (55, 79, 80). This allows sufficient recovery potential associated with perilesional reorganization in the adjacent cerebral tissue in response to various neurorehabilitative approaches.

**Table 1:** **Classification of ischemic brain infarcts**.

Type	Infarct location	Pathogenesis	Response to thrombolysis
**I**	**Territorial**	**Occlusion of cerebral artery branch**	
I.1	Cortical	Distal branch	Early
I.2	Cortico-subcortical	Proximal branch	Limited
**II**	**Striatocapsular**	**Occlusion of MCA stem**	
II.1	±Insula	Infarct core	Early
II.2	+Periventricular white matter	Large lesion	Limited
**III**		**Lacunar hyalinosis of arterioles**	Limited
III.1	Fiber tracts		
III.2	Internal capsule (anterior choroidal artery)		
III.3	Basal ganglia, lateral thalamus		
III.4	Medial and anterior thalamus (perforating branches of posterior cerebral artery)		
**IV**		**Chronic hemodynamic deficit + downstream emboli**	
IV.1	Cortico-subcortical	Extracranial artery occlusion ± intracranial large artery occlusion ± accompanied by reactive vasodilation	Limited
IV.2	Arterial borderzone	Extracranial artery occlusion	

*Adapted from Seitz and Donnan (75)*.

Ischemic lesions of large parts of or the entire striatocapsular region typically result from an embolic occlusion of the MCA stem (81) (Table 1). If reperfusion is achieved early, only the deep perforating arteries and the arteries that supply the insular cortex may remain obstructed causing infarcts of the lentiform nucleus and insula (82). However, when collaterals are insufficient due to arteriosclerotic changes in multiple cerebral arteries (41, 53, 54), the infarct lesions become larger involving to a larger extent also the hemispheric white matter. This causes hemispatial neglect and conduction aphasia due to cortico-cortical and cortico-subcortical disconnections (62, 83, 84).

Small-sized, lacunar-type, infarcts (Type III infarcts) result from an occlusion of the small penetrating cerebral arteries or even arterioles. They typically occur in the anterior choroidal artery, the deep perforating lenticular MCA branches, the thalamic branches of the PCA, or in brainstem structures and the pons (85, 86). In spite of their small spatial dimension, but due to their strategic location, they cause well-defined neurological syndromes, such as pure motor and pure sensory stroke (Table 1). These infarcts have a limited recovery potential as predicted by a loss of motor-evoked potentials and asymmetry of water diffusivity on MR imaging (55, 87, 88). The crucial role of the white matter for functional outcome becomes apparent from the observation that small infarcts in the precentral gyrus allow for profound motor recovery, whereas infarcts of similar volume in the periventricular white matter or the internal capsule may induce a severe and persistent hemiparesis (89, 90). Interestingly, white matter damage in stroke was found in a large genome-wide association study to be related to a mutation in chromosome 17 (91).

Patients with a chronic occlusion of extracranial cerebral arteries resulting from dissection or long-standing cerebrovascular disease constitute Type IV infarcts (Table 1). These patients may become symptomatic with transient ischemic attacks due to small embolic or hemodynamically induced watershed infarcts in cerebral white matter (92, 93). In these patients, blood flow depression induces a reactive vasodilatation of the intracranial blood vessels resulting in a severe delay in cerebral brain perfusion in the presence of an enhanced cerebral blood volume (94, 95).

### Perilesional Plasticity

Ischemia and reperfusion evoke a large number of biochemical, metabolic, and immunological processes that evolve sequentially as identified in animal experiments (96). In addition, there are rapid changes in the expression of genes, neurotransmitters, such as glutamate and GABA, as well as neurotrophic mediators implicated as molecular substrates related to perilesional reorganization (21, 97–101). These biochemical changes are accompanied on the microscopical level by the growing of axons and formation of new synapses in the perilesional vicinity and in remote locations in functionally related areas in the affected and contralesional “non-affected” hemisphere (102, 103). In particular, they occur when animals recover in an enriched environment or are subjected to dedicated training (104, 105).

Non-invasive brain stimulation techniques have provided means to explore changes of cortical excitability following stroke in humans. There are different technical approaches that allow to enhance or to suppress brain activity (106). By these methods, diagnostic and therapeutic goals were aimed for as summarized in Table 2. For example, using paired-pulse TMS, it was found that within the first 7 days after a brain infarct, there is an enhanced cortical excitability in the cortex adjacent to the brain lesion (107–109). In fact, the sites of residual motor representation move into the region of maximal cortical disinhibition (110). Also, fMRI activation areas related to finger movements were found to remap to spared more dorsal locations of the motor cortex (111, 112). Notably, an enhanced excitability was propagated to the contralesional hemisphere (14, 107–109, 113). It decreased in the patients who showed a good recovery within the 90 days, while it persisted in those patients with poor recovery (114). In keeping with these observations, functional MRI performed ~2 days after stroke revealed an area in the ipsilesional postcentral gyrus and posterior cingulate gyrus that correlated with motor recovery ~3 months after stroke (115). Conversely, recovery of hand function was associated with progressively lateralized activation of the affected sensorimotor cortex (116–118).

**Table 2:** **Techniques, actions, and effects of non-invasive stimulation of the human brain**.

Transcranial magnetic stimulation (TMS)				Transcranial electrical stimulation		
		**Neuromodulatory effects**				
		
Single pulse TMS	Paired-pulse TMS	Repetitive TMS	Patterned rTMS	Direct current stimulation tDCS	Alternating current stimulation	Random noise stimulation
	Intracortical (single coil)	1 Hz TMS (inhibitory)	Continuous theta-burst stimulation (inhibitory)	Cathodal tDCS		
	Cortico-cortical (two coils)	>4 Hz TMS (excitatory)	Intermittent theta-burst stimulation (excitatory)	Anodal tDCS		

*After Liew et al. (119)*.

Non-invasive electrical anodal stimulation of the affected motor cortex was found to augment motor skill acquisition due to improved consolidation but not due to long-term retention of the task (120). In contrast, application of 1-Hz repetitive TMS (rTMS) that downregulates the contralesional motor cortex improved the kinematics of finger and grasp movements in the affected hand (121). This was accompanied by an overactivity in the contralesional motor and premotor cortical areas predicting improvement in movement kinematics. One may wonder if long-term retention of the induced effects can be achieved by longer lasting stimulation or by the combination of voluntary action and direct brain stimulation preferentially in the acute phase after stroke. The combination of electrical stimulation of finger extensor muscles and training over 2–3 weeks did not result in a greater improvement of dexterity of the affected hand as assessed with the Jebson test than each intervention alone (122). Subjects with an intact motor cortex showed a greater improvement than those who had damage of the motor cortex. Similarly, in chronic stroke-induced aphasia rTMS over the left inferior frontal gyrus resulted in an increase of reaction time or error rate in a semantic task suggesting restoration of a perilesional tissue in the left hemisphere after stroke (123, 124). Given the human postlesional changes of cortical excitability it may be intriguing to rebalance the interhemispheric rivalry by direct cortical stimulation or peripheral stimulation (125–128). An even greater effect was observed when bihemispheric direct cortical stimulation was used to activate the affected motor cortex and to inhibit the contralesional motor cortex (129). Cortical stimulation in association with motor training also improved motor performance (128, 130–132). Along the same line, combining peripheral nerve stimulation to the affected hand with anodal direct current stimulation of the affected motor cortex in chronic stroke facilitates motor performance beyond levels reached with either intervention alone (133).

### Infarct Induced Damage to Cortico-Cortical and Cortico-Subcortical Connections

**Figure 2:**
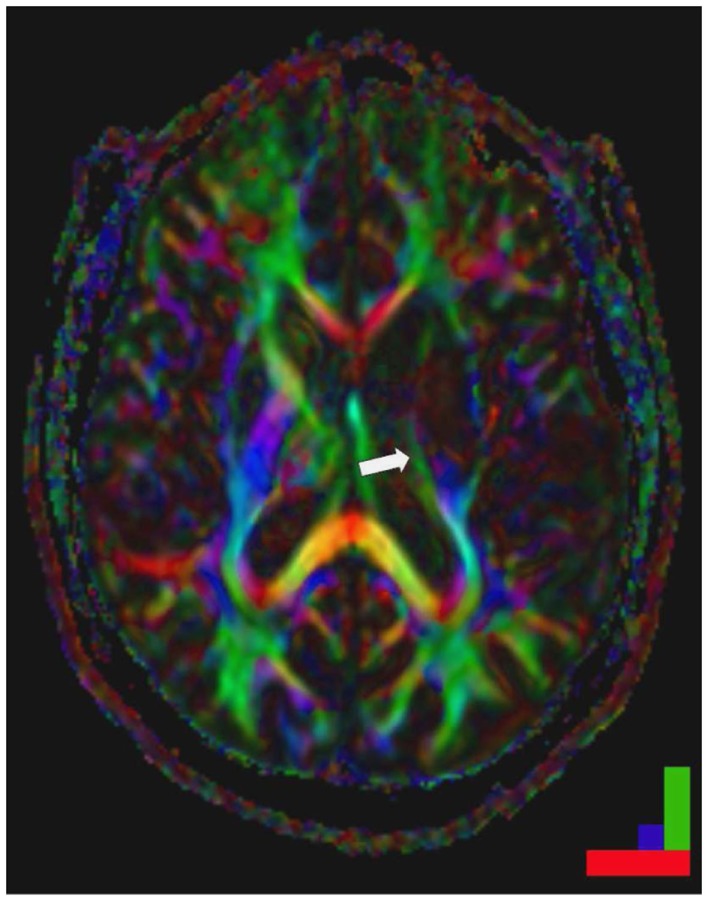
**Striatocapsular stroke (Type II.1) in a patient with persistent hemiplegia**. Note the small but complete destruction of the posterior limb of the internal capsule (arrow). Color bar: green fronto-occipital diffusion, red right-left diffusion, blue dorso-ventral diffusion. By permission of Oxford University Press (URL www.oup.com), Free permission Author reusing own material, p. 82 fig: 6.4 (left part) from “Stroke Rehabilitation” edited by Carey and Leeanne (140).

Corticospinal fibers are key factors for the recovery of motor function after stroke as demonstrated with different imaging modalities as well as electrophysiological measures (55, 87, 134–136). In non-human primates, the cortico-reticulo-spinal and cortico-rubro-spinal tracts are known to mediate motor functions in case of corticospinal tract lesions (137, 138), since these tracts have been described as functionally redundant in healthy animals (139). In humans, however the corticospinal tract is of key relevance for motor recovery (Figure 2). In fact, the integrity of the corticospinal tract determines the movement related motor cortex activation (65, 87). When there are no motor evoked potentials and there is poor recovery in chronic patients, the fractional anisotropy of the posterior part of the internal capsule as assessed by diffusion tensor imaging was altered in the affected hemisphere (68, 87). Notably, these patients had bilateral fMRI activations in relation to finger movements, while in the patients with a lower asymmetry, there was an activation lateralized to the affected hemisphere.

There are not only changes in the efferent motor fiber tracts but also in the cortico-cortical and probably also cortico-subcortical fiber tract systems during recovery. In fact, the intracortical excitability as assessed with TMS was increased in motor cortex of both hemispheres both in subcortical and cortical infarcts (108, 114, 141, 142). Conversely, ipsilesional MEPs were more easily elicited from proximal muscles in stroke patients than in healthy subjects (143–145). Moreover, motor cortical connectivity was shown by diffusion tensor imaging to be enhanced after stroke (146). Additionally, orientation uncertainty and greater white matter complexity correlated with functional outcome and were possibly triggered by functional demands (146, 147). In addition, it was found recently that the pyramidal tract splits up in the pons forming a ventral and a dorsal tract. When both tracts are affected, patients have a poor recovery, while continuity of the projections in the dorsal portion was characterized by good recovery (136). In addition, in chronic stroke patients, DTI-derived measures of transcallosal motor fibers as well as ipsilesional corticospinal tracts pyramidal tract and alternate fiber tract determine the therapeutic response to rehabilitation. The more the diffusivity profiles resembled those observed in healthy subjects, the greater a patient’s potential for functional recovery (88). These findings accord with the evidence from functional imaging suggesting that the concerted action of both cerebral hemispheres is required for recovery. This corresponds well to the observation that even patients with an excellent recovery may show a bilateral activation pattern (148, 149). This abnormal activity involved premotor cortical areas and was largely reminiscent of activity patterns in learning but are essentially transient in nature (84, 115, 149). Notably, tiny activation areas in contralesional motor cortex were related to mirror movements that frequently occur initially after stroke (150).

Network types of neuroimging data analysis have revealed that there is a pathological interhemispheric interaction between the ipsi- and contralesional motor cortex as well as between the ipsilesional supplementary motor area (SMA) and contralesional motor cortex in patients with a single infarct lesion (151, 152). In unilateral movements of the affected hand, there was an inhibitory influence from the contralesional to the ipsilesional motor cortex which correlated with the degree of motor impairment (152). In bimanual movements, the interaction of the ipsilesional SMA and the contralesional motor cortex was reduced, and this correlated with impaired bimanual performance. This can be related to the observation that there was less activation in contralesional motor cortex when the motor task did not require working memory demands and no change when the task required online visual feedback monitoring (153). Furthermore, connectivity strength of the prefrontal cortex to the premotor cortex was enhanced in relation to motor imagery highlighting its role for higher order planning of movement (154).

## Disease-Related Limitations of the Recovery Potential

### Associated Diseases

It has been known for 30 years that patients with acute stroke may develop cognitive impairment and mood disorders which may aggravate their clinical conditions (155, 156). However, only recently it was shown in a large database of stroke patients subjected to systemic thrombolysis that the pre-existing functional impairment may reduce the patients’ response to thrombolysis and the survival rate (157). In a prospective, open label study of 192 patients (68 ± 13 years, 50% males) subjected to intravenous thrombolysis the patients was found to improve (*P* < 0.0001), while 18% deceased within 100 days (158). This was predicted by older age (76 ± 10 years, *P* < 0.05) and more severe affection on admission (*P* < 0.0001). Also, these patients more frequently had atrial fibrillation (*P* < 0.03) than the surviving patients. Furthermore, it was found that stroke patients with a severe prestroke disability have a virtually 50% risk of deceasing. It seems that women are particularly liable of depression after stroke and that this is related to a greater stroke severity (159). Of note are patients with migraine that to a large proportion suffer from small vessel disease (160) or hemorraghic stroke (161). This is of great functional relevance since white matter disease due to small vessel disease enhances the risk of depression, physical disability, and a reduction of quality of life (162). Furthermore, there is evidence from a huge meta-analysis that ischemic stroke is associated with the presence and subsequent development of dementia, particularly in recurring ischemic stroke (163). In addition, dementia was found to be associated with increased letality (164). Interestingly, small vessel disease is the most frequent vascular abnormality in patients with Parkinson’s disease (165, 166). These vascular changes seem to predispose patients with Parkinson’s disease to cerebrovascular accidents (167). Arteriosclerosis was found to be of particular relevance for Parkinsonian gait, while macroscopical infarcts seem to result in rigidity (168). Moreover, infarcts induce epileptic seizures (169), which may mimic stroke as in Todd’s paresis and impair recovery due to reduced consciousness. Beyond that stroke may induce changes of affect including alexithymia (58) or depression (170). The latter was found to be most severe in chronic obstructive pulmonary disease, smoking, and in patients with poor socioeconomic status. Also the increasing lesion load with recurrent strokes in the elderly may predispose to depression (171) and death (172). Thus, there is an intimate interaction of stroke and comorbities the latter of which impair the recovery potential of stroke patients. Deeper insight into the pathophysiology of these interactions is required to counteract these detrimental effects and to enhance the recovery potential of the multimorbid stroke patients.

### Functional Deficits in Brain Infarcts

**Figure 3:**
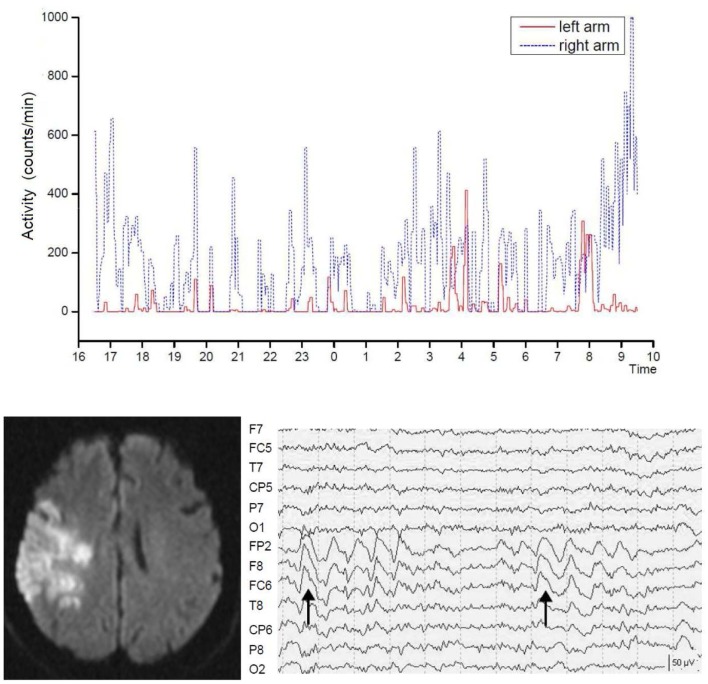
**Severely reduced spontaneous movement activity in the affected left arm in right hemispheric brain infarct**. Shown is the recording time between 4 p.m. until 10 a.m. the following day. The intermittent slow wave activity in electroencephalographic recordings predicted poor motor recovery. Dotted lines indicate seconds. From Ruan and Seitz (174).

The neurological deficit has two expressions. There is the impairment to perform actions on command which is usually assessed in clinical examinations. And there is the decrease in spontaneous motor activity which may be functionally relevant (Figure 3). In a prospective study of 25 patients (63 ± 10 years) with acute MCA stroke and seven control patients without neurological disease (61 ± 14 years), movement activity was measured continuously for 4 days in both arms using Actiwatches (Cambridge Research Instruments, UK). Stroke patients with an initial decline in arm movement activity showed no increase in movement activity in either arm over 4 days after stroke, while other patients improved steadily after admission. The impairment continued to be different among the two groups 3 months after stroke (173). Stroke severity, location and treatment, as well as arterial blood pressure and body temperature were not different among the groups. But, in the non-recovering patients, the C-reactive protein was elevated and related to a low number of waking hours. These results support the notion that in the acute stage after MCA stroke, there are patients with a secondary decline in general motor activity and an enhanced sleep demand which was related to systemic inflammation.

Moreover, recordings with the electroencephalogram (EEG) revealed that stroke patients may exhibit focal slow wave activity (SWA) as well as focal epileptic changes in the affected hemisphere (175–177). Focal SWA (1–4 Hz) has been reported to predict poor recovery from stroke (178–180) but can last even for years (181). Notably, EEG recordings have revealed that, in addition to their neurological deficit, stroke patients also have an abnormal sleep architecture (182, 183). It is unclear, however, what the functional impact of SWA is on spontaneous movement activity of the affected side after stroke. In fact, stroke patients with similar infarcts concerning lesion location and volume may show recovery patterns of the formal neurological assessment that are not reflected by the spontaneous movement activity of the affected limbs (184, 185). In acute stroke patients (68 ± 8 years) and age-matched controls (68 ± 12 years), movement activity was measured continuously and synchronously with the EEG for 24 h in both arms using actiwatches (174). The stroke patients had lower total sleep time (*P* = 0.031), sleep efficiency (*P* = 0.019), percent non-rapid eyement movement sleep (*P* = 0.034), and percent sleep stage N2 (*P* = 0.003) and showed reduced spontaneous movement activity in the affected arm during wakefulness. Stroke patients with abnormal focal SWA showed less spontaneous arm movement activity than those without SWA, while there were no differences in the sleep parameters (Figure 3). These findings accord with earlier observations by Bassetti and Aldrich (175) supporting the notion that sleep architecture is impaired in stroke patients leading to sleep fragmentation, increased wakefulness, and increased REM latency (186). Furthermore, the stroke patients with SWAs enjoyed a limited recovery as assessed with the NIHSS. Thus, focal SWA is a marker of profound brain pathology.

### Times-Lines for Post-Stroke Recovery

The neurological deficits can regress substantially in the early period after ischemic stroke following acute stroke treatment with arterial recanalization and effective reperfusion. The relatively early recovery in patients with small cortical lesions steadily evolves over weeks and levels out over the subsequent months (112, 187, 188). In contrast, the processes of cerebral re-organization are slow and may need many months to complete. In the acute phase of stroke, it is difficult to predict the degree of ultimate recovery, since there is a large heterogeneity of recovery over the first 3 months after stroke (12). Prediction becomes progressively better the more specific and differentiated the physiological assessment measures are and the longer the time since stroke (70, 189, 190). For example, the neurological state by day 4 predicts the long-term neurological outcome (188, 191). The recovery of activities of daily living usually develop within 26 weeks after the stroke insult and is often accompanied by compensatory hand use (192, 193).

### Neurorehabilitative Training

There are numerous reports about rehabilitative approaches to improve the neurological deficit following stroke (4, 13). Notably, patients older than 65 years benefit as much as younger patients from intensive rehabilitation (190, 194), while younger patients typically improve more on mobility, balance, walking, and grip strength (195). The intensity of the training rather than the type of training appears to determine long-term improvement of motor function (113, 196–198). While passive training of wrist movements was reported to be clinically effective and associated with change in cortical activation (199), volitional control of finger and thumb extensions was found to play an important role for successful hand shaping and grasping of objects (147, 214). Importantly, repetitive training of the affected arm resulted in an increase of activation in the sensorimotor cortex related to hand movements which initially persisted for weeks after training completion and then decreased in magnitude in relation to the functional gain (200, 201). In contrast, mirror therapy was found to improve the neurological status immediately after the intervention and to be effective even at long-term follow-up (202, 203).

Training of the affected limb as well as training targeting the non-affected limb has been proposed to be effective. For example, use of bilateral synergies has been reported to improve the motor capacity of the paretic arm (204). It was described that active–passive bilateral arm therapy can produce sustained improvements in upper limb motor function in chronic stroke patients. This was paralleled by an enhanced ipsilesional motor cortex excitability and an increased transcallosal inhibition from ipsilesional to contralesional motor cortex (205). Conversely, the concept of “learned non-use” was implemented in new approaches of rehabilitative strategies in chronic patients with brain infarction (206, 207). This therapy has been shown to be successful even when applied in the chronic state to moderately affected patients (65, 208, 209). This beneficial effect of constraint-induced movement therapy is likely to be composed of focusing the patient’s attention to the affected side and imposing repetitive training. It was shown to result in improved motor function and enhanced activation in the partially damaged sensorimotor cortex and other gray matter areas including the hippocampus (210).

**Figure 4:**
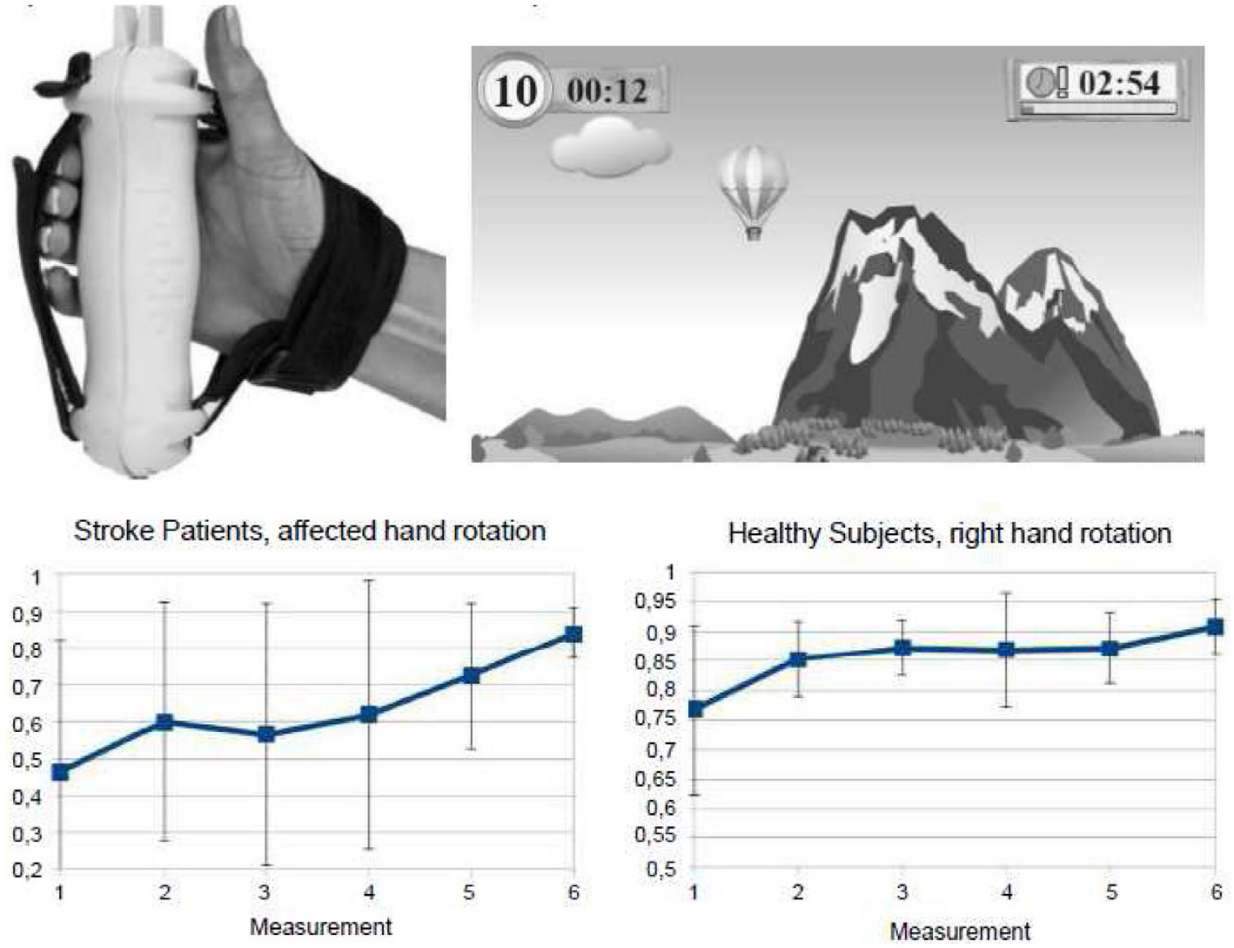
**Gaming-based training scenario using the commercially available hand hold PABLO^R^-device**. Hand movements are measured by acceleration and force sensors and thereby steer objects in virtual reality games. Training on consecutive days enlarged the angle of hand rotations and decreased the heterogeneity of movement execution both in healthy subjects and stroke patients. From Seitz et al. (213).

Recently, computer-based training approaches employing virtual realitiy scenarios have been developed for neurorehabilitative training purposes, since it was assumed that they engage the patients emotionally and thereby enhance their inclination to embrace rehabilitation training activities. For example, the rehabilitation gaming system (RGS) is a flexible, virtual reality-based device for rehabilitation of neurological patients (211). In fact, it was shown to effectively improve arm function in acute and chronic stroke patients. Furthermore, it was shown by fMRI that the RGS engages human mirror neuron mechanisms that underly visuomotor coordination (212). Similarly, the handhold multifunctional PABLO^R^-device was applied for the training of visuomotor-tracking paradigms. It was observed that training of the right dominant hand improved visuomotor coordination of hand rotation movements in both hands in healthy subjects. Notably, it was successful only in the trained hand in stroke patients (Figure 4). Since these gaming applications capitalize on the positive affect of the patients and engage brain structures known to be related to emotional processing (212), these approaches point into new avenues of post-stroke rehabilitation opening new frames for the recovery potential after stroke.

## Conflict of Interest Statement

The authors declare that the research was conducted in the absence of any commercial or financial relationships that could be construed as a potential conflict of interest.
